# A Phenomic Scan of the Norfolk Island Genetic Isolate Identifies a Major Pleiotropic Effect Locus Associated with Metabolic and Renal Disorder Markers

**DOI:** 10.1371/journal.pgen.1005593

**Published:** 2015-10-16

**Authors:** Miles C. Benton, Rodney A. Lea, Donia Macartney-Coxson, Michelle Hanna, David A. Eccles, Melanie A. Carless, Geoffrey K. Chambers, Claire Bellis, Harald H. Goring, Joanne E. Curran, Jacquie L. Harper, Gregory Gibson, John Blangero, Lyn R. Griffiths

**Affiliations:** 1 Genomics Research Centre, Institute of Biomedical Health and Innovation, Queensland University of Technology, Brisbane, Queensland, Australia; 2 Biomarkers Group, Kenepuru Science Centre, Institute of Environmental Science and Research, Wellington, New Zealand; 3 Texas Biomedical Research Institute, San Antonio, Texas, United States of America; 4 School of Biological Science, Victoria University of Wellington, Wellington, New Zealand; 5 Malaghan Institute of Medical Research, Wellington, New Zealand; 6 School of Biology, Georgia Institute of Technology, Atlanta, Georgia, United States of America; University of Tasmania, AUSTRALIA

## Abstract

Multiphenotype genome-wide association studies (GWAS) may reveal pleiotropic genes, which would remain undetected using single phenotype analyses. Analysis of large pedigrees offers the added advantage of more accurately assessing trait heritability, which can help prioritise genetically influenced phenotypes for GWAS analysis. In this study we performed a principal component analysis (PCA), heritability (h^2^) estimation and pedigree-based GWAS of 37 cardiovascular disease -related phenotypes in 330 related individuals forming a large pedigree from the Norfolk Island genetic isolate. PCA revealed 13 components explaining >75% of the total variance. Nine components yielded statistically significant h^2^ values ranging from 0.22 to 0.54 (P<0.05). The most heritable component was loaded with 7 phenotypic measures reflecting metabolic and renal dysfunction. A GWAS of this composite phenotype revealed statistically significant associations for 3 adjacent SNPs on chromosome 1p22.2 (P<1x10^-8^). These SNPs form a 42kb haplotype block and explain 11% of the genetic variance for this renal function phenotype. Replication analysis of the tagging SNP (rs1396315) in an independent US cohort supports the association (P = 0.000011). Blood transcript analysis showed 35 genes were associated with rs1396315 (P<0.05). Gene set enrichment analysis of these genes revealed the most enriched pathway was purine metabolism (P = 0.0015). Overall, our findings provide convincing evidence for a major pleiotropic effect locus on chromosome 1p22.2 influencing risk of renal dysfunction via purine metabolism pathways in the Norfolk Island population. Further studies are now warranted to interrogate the functional relevance of this locus in terms of renal pathology and cardiovascular disease risk.

## Author Summary

While many large genetic association studies have identified genes playing a role in complex disorders, there is still concern over the amount of missing genetic heritability. With this in mind, we have used a data reduction approach alongside pedigree-based association to obtain highly heritable components which explain 'hidden' variance of multiphenotypes within a large pedigree from the Norfolk Island genetic isolate. The most heritable of these components involved 7 traits reflecting metabolic and renal functionality, association of which locates to an intergenic region on chromosome 1p22.2. By integrating gene expression information, we identified enrichment of a purine metabolism pathway, further strengthening the metabolic nature of the observed association. Adding additional support to our approach, we show association of the tagging SNP (rs1396315) in an independent US population. The findings presented here are of particular interest as they implicate pleiotropic effect loci and newly associated biological pathways underlying cardiovascular disease risk.

## Introduction

Cardiovascular diseases (CVD) are highly prevalent conditions and are the leading cause of morbidity and mortality in many developed nations [1]. In general, CVDs are comorbid conditions that arise from several major underlying risk factor traits, e.g. excess adiposity, hypertension and dyslipidemia. These component traits, and therefore risk of CVD, are influenced to varying degrees by inherited genetic factors. Many genome-wide association studies (GWASs) have been conducted in recent years and have identified common genetic variants associated with all major risk factor traits for CVD.

It is well known that risk factor traits for CVD tend to be correlated in populations suggesting the presence of underlying genetic variants that affect multiple different phenotypes, i.e. exert pleiotropic effects. GWAS analysis aimed at multiple CVD phenotypes simultaneously may reveal such genetic loci, which could remain undetected using univariate (single) phenotype analysis methods. For example, a large-scale population-based GWAS of >25,000 Americans revealed previously unrecognised variants within *APOC1*, *BRAP* and *PLCG1* that may confer pleiotropic effects on composite traits associated with CVD [2]. More recently, a GWAS meta-analysis of 85,500 subjects identified 25 variants with pleiotropic associations to metabolic syndrome traits [3]. These results support a multiple phenotype (“phenomics” based approach) for mapping pleiotropic effect genes for CVD.

GWAS analysis of isolated populations have also been successfully utilised to map genes for CVD traits [4–6]. Features such as founder effect, reduced genetic and environmental diversity, as well as the availability of very large multi-generational pedigrees can offer advantages over general population GWAS approaches [4]. For example, pedigree information can allow accurate estimates of heritability to be calculated that can help prioritise genetically influenced phenotypes for GWAS analysis. Moreover, founder effect leading to reduced genetic heterogeneity in isolated populations can amplify genetic effects and thus improve power to detect susceptibility loci [7,8].

The Norfolk Island Health Study (NIHS) is a long running investigation of the genetics of CVD traits in the isolated population of Norfolk Island—situated off the Australian East Coast [9]. The Norfolk Island population was founded by a small group of European “Bounty” mutineers and their Polynesian wives in the late 1700s and now forms a 6000-member pedigree spanning 11 generations [10–12]. The Norfolk Island population exhibits features such as genetic founder effect and admixture [13], and has high rates of heritable CVD traits such as hypertension and obesity [9,14]. Recently, we completed pedigree-based GWASs of 37 CVD-related traits in the Norfolk pedigree and identified a number of loci to be associated with individual phenotypes (under review). The aim of this new study was to perform a multi-phenotype (or phenomics-based) GWAS in the genetic isolate of Norfolk Island in an effort to identify novel pleiotropic effect loci influencing CVD related traits.

## Results

This study focused on a well-characterised core pedigree from the Norfolk Island genetic isolate [14]. This core-pedigree is comprised of 330 individuals and has been measured for 37 quantitative traits as part of the NIHS to assess the genetics of CVD. The study involved principal component analysis (PCA) of all 37 traits to assess inter-correlation, and subsequent heritability (h^2^) analysis to estimate genetic variance of resultant components. This was followed by pedigree-based GWAS of heritable components using SNPs to identify major pleiotropic effect loci for CVD risk phenotypes in this population.

### Principle Component Analysis (PCA) of CVD traits

**Fig 1:**
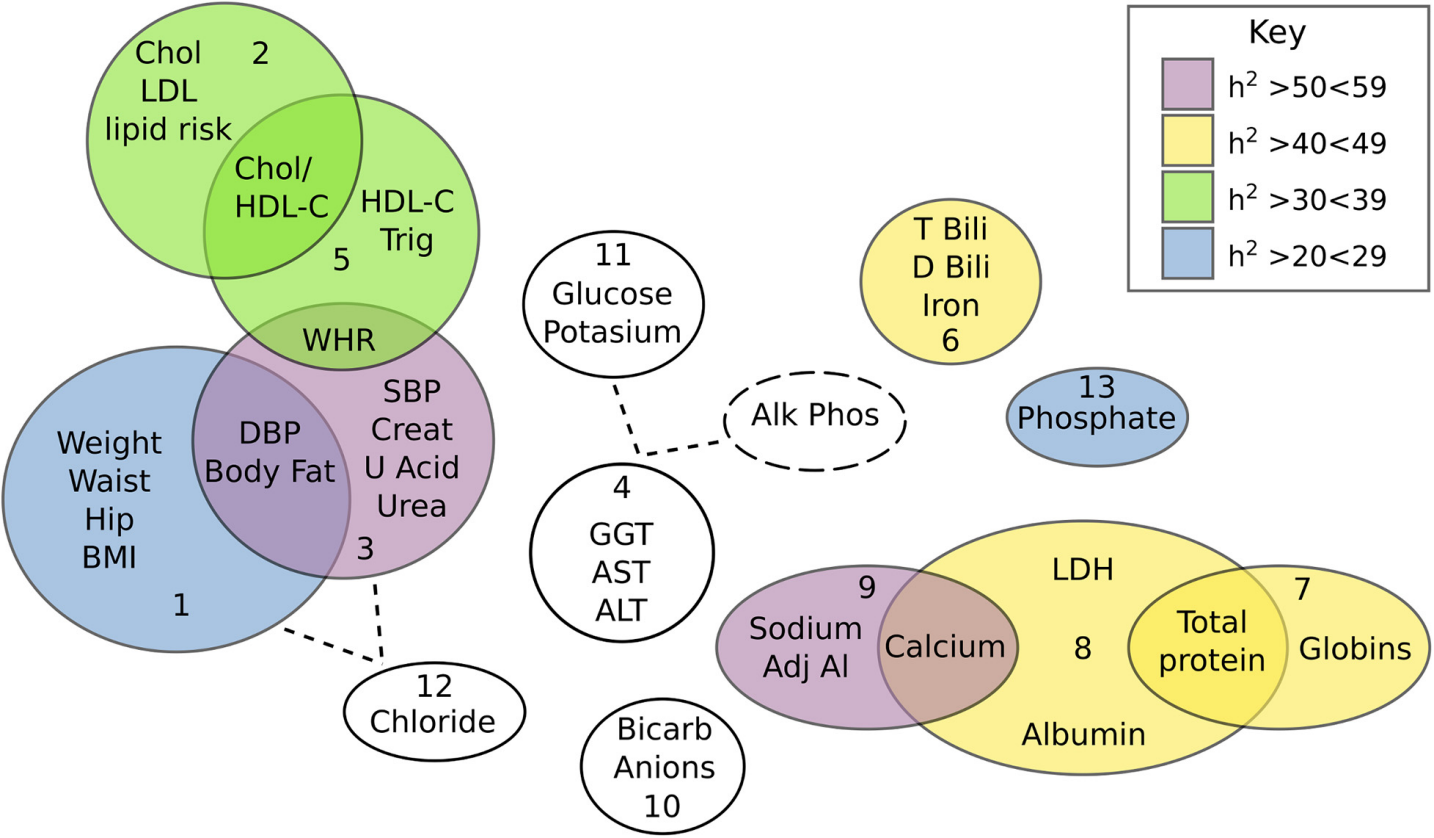
Venn diagram showing the inter-correlation and heritabilities of components extracted from the PCA of 37 quantitative traits. Relationships between components were inferred either by variable overlap, or loadings on a component greater than 0.35. Heritability of each component is colour coded (as per key) with the non-significant traits being coloured white.

An ‘unsupervised’ PCA of 37 CVD related traits was conducted (trait summaries listed in S1 Table). The PCA extracted 13 composite phenotypes which together explained >75% of the total variance (Table 1). Trait loading scores for each of these components is available in S2 Table. Heritability values for all 13 components, as well as a total combined component were calculated. Nine components showed nominal significance with heritability estimates ranging from 0.22–0.55 (*P*<0.05). After correction for multiple testing was applied only Component 3 and Component 9 remained significant (P<0.0038). Component 3 was the most heritable (h^2^ = 0.55) and was loaded with 7 measures related to CVD—% body fat, waist-to-hip ratio, systolic and diastolic blood pressure, creatinine, blood urea nitrogen, and uric acid. The measures most strongly correlated with Component 3 were blood urea nitrogen (0.76), creatinine (0.74) and uric acid (0.61) which are all kidney function markers suggesting that the Component 3 phenotype may represent renal dysfunction. The relationship between all extracted components is displayed in Fig 1. Overlap between components is suggestive of co-morbidity among traits as well as the possibility of genetic commonalities. The clinical relevance of the Component 3 score was explored by comparison with the formal Framingham CVD score [15] and showed a positive correlation (r = 0.4, P = 1.2x10^-8^).

**Table 1:** PCA and heritability analysis of 37 quantitative traits.

Component	Loaded Traits	Eigenvalue	% Variance	Cumulative%	h^2^	P value
Component 1	body fat, weight, waist, waist circumference, BMI, hip circumference, DBP	7.84	21.19	21.19	0.27^†^	2.35e-2
Component 2	cholesterol, LDL,	2.96	8.00	29.19	0.37^†^	4.07e-3
Component 3	body fat, WHR, SBP, DBP, creatinine, urea, uric acid	2.60	7.02	36.22	0.55^†^	2.85e-5^‡^
Component 4*	GGT, ALT, AST (liver enzymes)	2.08	5.63	41.85	0.22	5.66e-2
Component 5	triglyceride’s, HDL, WHR, chol/HDL ratio	1.92	5.19	47.04	0.32^†^	1.43e-2
Component 6*	iron, total bilirubin, direct bilirubin	1.80	4.85	51.89	0.40^†^	2.65e-2
Component 7	total protein, globin	1.57	4.23	56.12	0.46^†^	9.08e-3
Component 8*	LDH, albumin, calcium, total protein	1.42	3.83	59.95	0.41^†^	2.50e-2
Component 9*	Sodium, calcium, adj_al	1.39	3.75	63.70	0.53^†^	1.26e-4^‡^
Component 10*	bicarb, anions	1.16	3.12	66.82	0.14	1.18e-1
Component 11*	glucose, potassium	1.14	3.08	69.91	0.08	3.33e-1
Component 12*	chloride	1.05	2.83	72.73	0.02	4.35e-1
Component 13*	phosphate	1.00	2.70	75.43	0.22^†^	3.99e-2
Component total*	(all components)	27.91	75.43	75.43	0.23	7.15e-2

*component was normalised (log transformed in SOLAR) due to large kurtosis

^†^h^2^ significant below nominal P<0.05

^‡^h^2^ significant after correction for multiple testing (P<0.0038)

### GWAS of principle components

**Fig 2:**
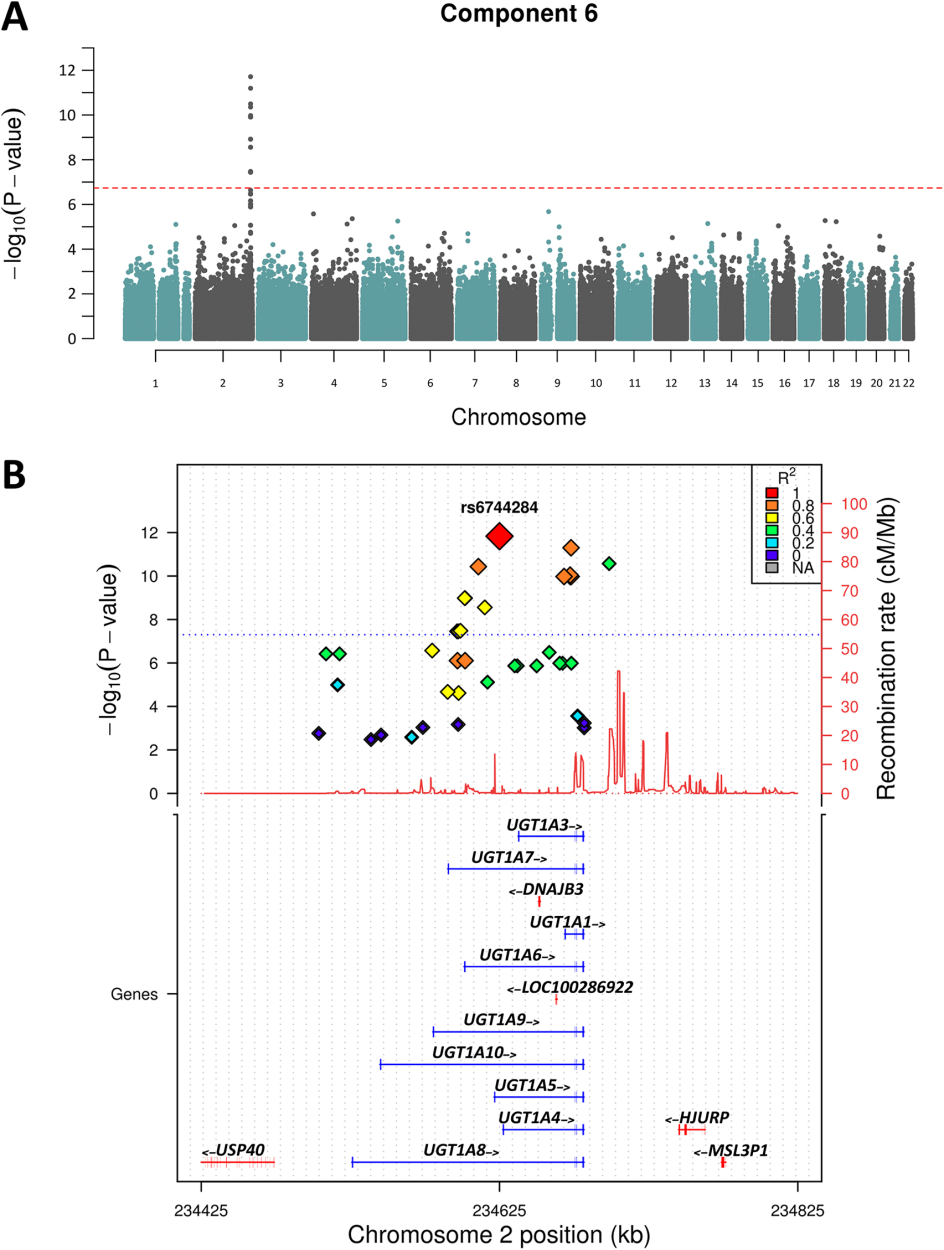
Manhattan plot of Chromosome 2 showing Component 6 associations. A) shows the genome-wide associations statistics with a distinct peak on chromosome 2 for bilirubin, B) refines the location of the peak to chr2q37.1, a region containing the UDP-glucuronosyltransferase gene family well known to metabolise bilirubin (UCSC track demonstrates the numerous isoforms). The blue dotted line indicates genome-wide significance level.

Following the PCA, we performed GWAS analysis for the 9 nominally significantly (p<0.05) heritable components shown in Table 1. We decided to test all heritable components regardless of multiple testing to explore the potential biological relevance of association hits. Of the 9 components only 2 showed association peaks with clusters of SNPs that exceeded the genome-wide statistical significance threshold (P = 1.8x10^-7^). Fig 2A shows that Component 6—loaded with iron and bilirubin measures—yielded the strongest association peak on chr 2q37.1 (P = 1x10^-11^). Analysis of the individual component traits revealed that this peak was entirely associated with total and direct bilirubin, and maps to the well-known bilirubin metabolising gene—*UGT1A1* [16–18], Fig 2B.

**Fig 3:**
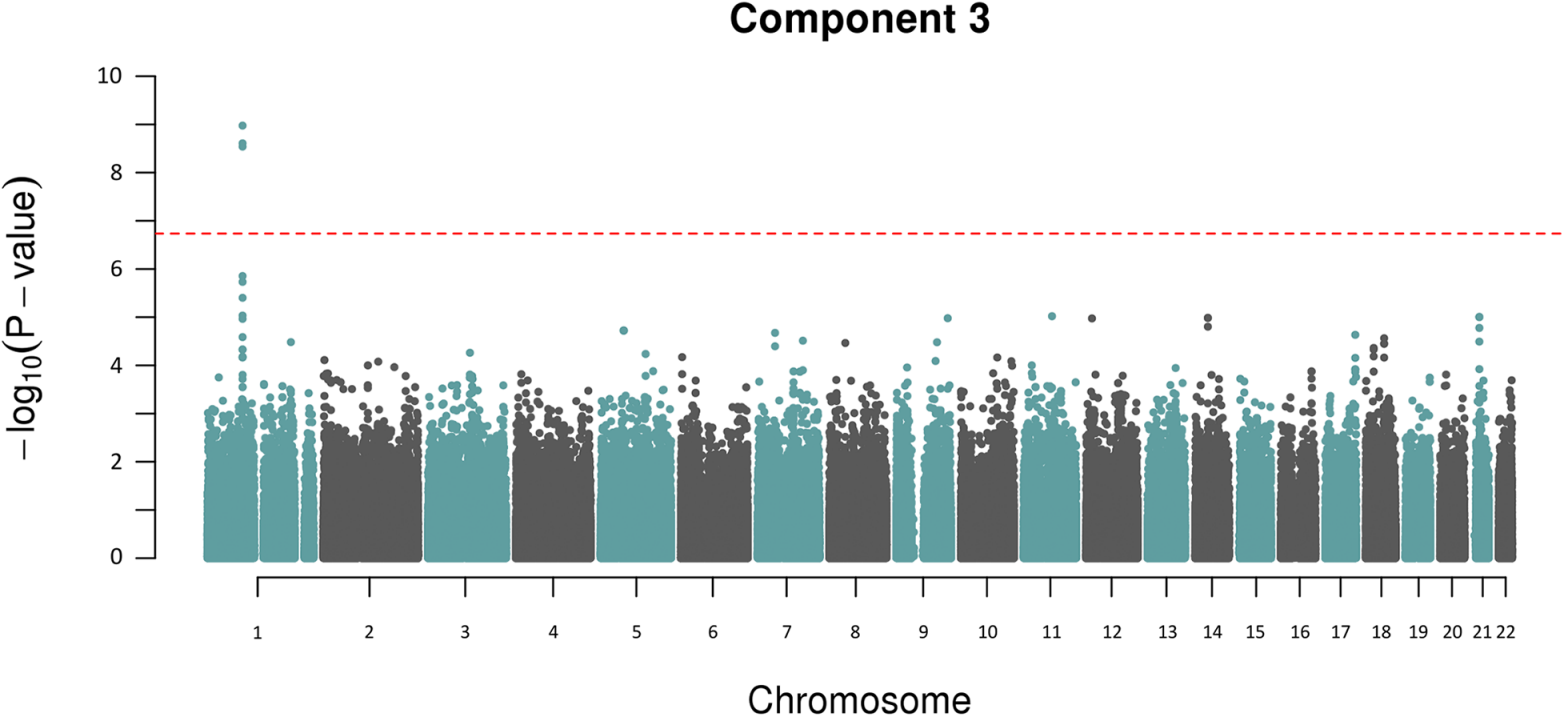
Manhattan plot for Component 3 showing an association peak on chr 1p22.2. The red dotted line indicates the study-wide *M*_*eff*_ significance threshold.

The other component trait to yield a statistically significant association peak was the most heritable component, Component 3. Fig 3 shows that the GWAS of Component 3 yielded three associated SNPs located at chr 1p22.2 (min P = 1.3x10^-9^ for rs1396315), as well as a supportive peak below the threshold of study-wide significance. GWAS of each of the 7 traits loaded into Component 3 individually did not show any significant peak at this locus (S1 Fig). Therefore, unlike Component 6 (explained by bilirubin alone) the Component 3 hit indicated a potential pleotropic effect locus that was further investigated.

**Fig 4:**
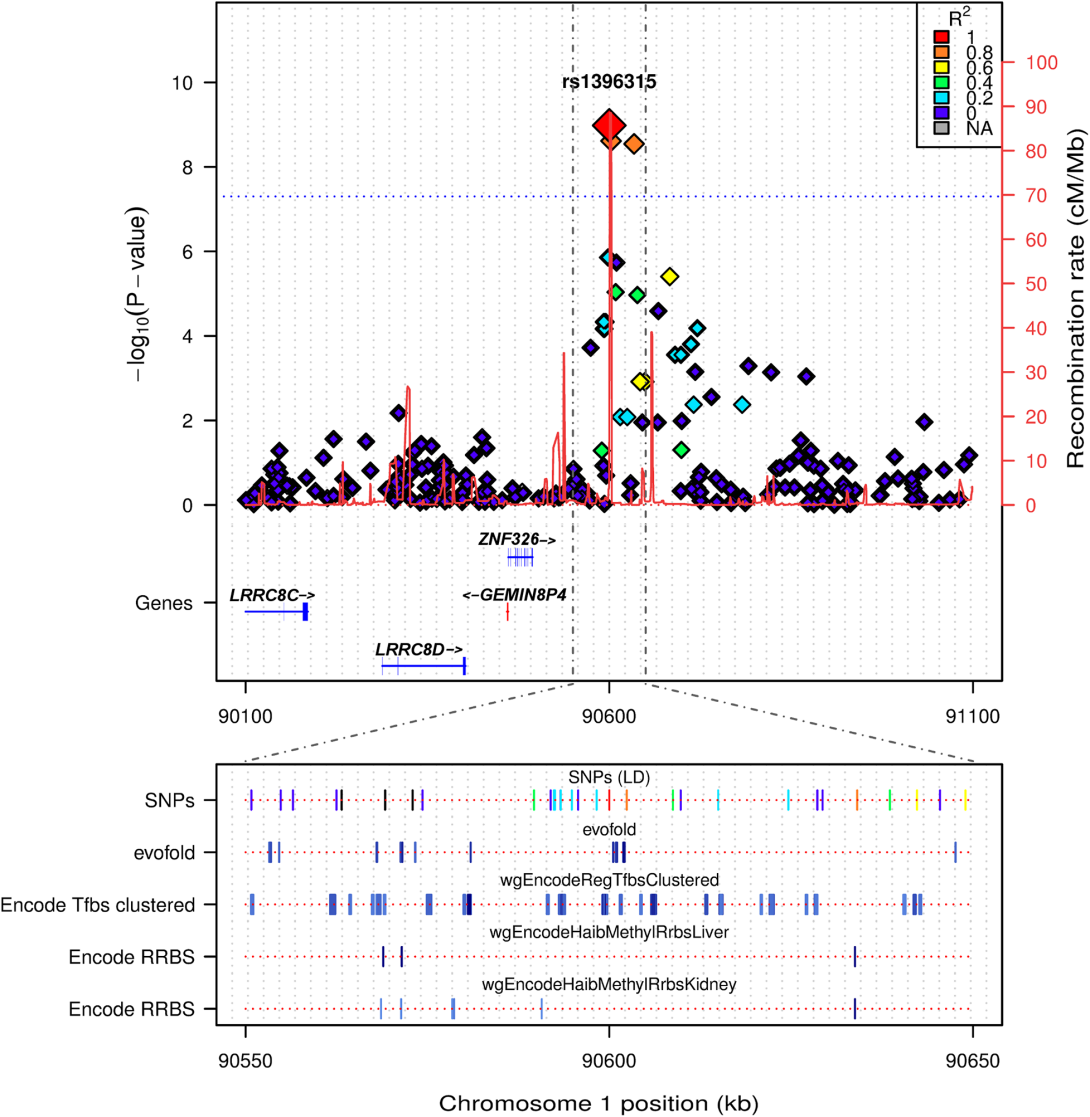
LocusTrack plot showing detailed annotation information of the associated chr1p22.2 region. The significance level of association is given on the left y axis (-log_10_(p-value)), while genomic recombination is displayed on the right y axis. Pairwise LD between the tagging SNP and each other SNP is indicated by colour. In the bottom panel several relevant tracks of UCSC data are provided, including: evofold RNA loop prediction; ENCODE transcription factor binding sites from ChIP-SEQ, and tissue specific RRBS methylation information from liver and kidney (both relevant tissue types for the identified phenotype).

To determine if there were any other additional independently associated variants in this region we performed an association analysis conditioning on rs1396315. This revealed no further associations indicating that only a single locus and resultant haplotype for Component 3 is present in this region. Linkage disequilibrium (LD) analysis of the SNPs spanning the Component 3 peak on chr 1p22.2 illustrated that rs1396315 tagged the associated haplotype and could be used as the reference SNP for further analysis (Fig 4). The allele frequency of the rarer allele (C) in the Norfolk pedigree was 0.24, which is consistent with the frequency in European populations [19]. Genotype association analysis of rs1396315 against the Component 3 score showed that the rare allele (C) was associated with elevated Component 3 score in an additive fashion. Locus-specific heritability estimates showed that this haplotype explained ~11% of the variation in the Component 3 score, indicating this is a major effect locus in the Norfolk Island population. Association analysis of rs1396315 against the individual phenotypes comprising Component 3 indicated that the strongest association was with blood urea nitrogen (P = 0.00006), followed by creatinine (P = 0.003) and uric acid (P = 0.005). The other traits loaded into Component 3 were not associated with rs1396315 when analysed independently (P>0.1). This indicates that the association of this SNP with Component 3 is primarily due to blood urea nitrogen, creatinine and uric acid—potential metabolic and renal functional markers.

The region directly surrounding the association peak on chr 1p22.2 did not reveal any known genes, with the closest, *ZNF326*, being ~325kb upstream (Fig 4). This led us to perform a detailed annotation survey of the region surrounding the peak on chr 1p22.2 using LocusTrack [20]. LocusTrack allows the incorporation of association statistics, SNP LD information, as well as several relevant UCSC annotation tracks, namely: evofold (bioinformatic prediction of RNA loop folding); ENCODE ChIP-SEQ transcription factor binding sites, and tissue specific methylation for liver and kidney (phenotypically relevant tissues) derived from ENCODE Reduced Representation Bisulfite Sequencing (RRBS). Fig 4 shows the chr 1p22.2 region of interest and all the relevant surrounding annotation information. It is interesting to note that while there may not be any genes directly annotated in the region, it does appear that there are distinct areas of potential control, i.e. transcription factor binding sites and tissue specific methylation, as indicated by the multiple UCSC tracks. Another annotated track of interest was that of the QTLs mapped from the rat genome database (RGB) to the human genome. There are 4 QTL specifically mapped to the area of interest at 1p22.2, all related to metabolic and renal dysfunction: Serum renin concentration QTL 2 (RGD:1300114); Non-insulin dependent diabetes mellitus QTL 15 and 20 (RGD:71115, RGD:70204), and Renal disease susceptibility QTL 4 (RGD:619619). Additional annotation is available for the chr 1p22.2 region in the form of a UCSC plot (S2 Fig), which contains the RGD QTL loci, and region specific information on DNase hypersensitivity clusters, as well as listing the individual transcription factor binding sites identified by ENCODE ChIP-SEQ. Finally, it is interesting to note that the association peak appears to lie on top of a region of increased genomic recombination (Fig 4).

### Replication analysis

To investigate replication of the association signal seen for rs1396315 with Component 3 we utilised; a) an outgroup of the Norfolk Island cohort (n = 375) who are genetically unrelated (independent) to the core pedigree and for whom comparable genotype and phenotype data was available (S3 Table), and b) a completely independent cohort of 738 individuals from a general United States of America (USA) population (the CHDWB cohort) [21]. This cohort had measurements available for 6 out of the 7 traits loaded into Component 3 (uric acid was not available) as well as genotype data for rs1396315.

Due to the difficulties of finding an independent replication cohort with the exact same set of 37 traits with which to replicate the PCA and generate Component 3 scores we had to explore other options for replication. We decided to use a linear modelling approach to attempt to predict individual Component 3 scores using only the 7 most heavily loaded traits for that component from the discovery phase. The linear model as generated in the original Norfolk Island discovery cohort is (all equations are as implemented in base R):

$$fit_{1}=lm\left(COMP3\;\sim \;WHR+BF+SBP+DBP+CREAT+UREA+U\_ACID\right)$$

The values from this model showed a very strong correlation with the original Component 3 scores and provided excellent predictive value (R^2^ 0.92, p < 2.16x10^-16^). The model (*fit*_*1*_) was used to predict Component 3 scores in the n = 375 unrelated Norfolk Island outgroup using the 7 traits of interest. The regression equation required slight modification for the US replication cohort because uric acid measures were missing. To do this we tested the effects of dropping uric acid on the overall linear model and observed a minimal reduction in predictive value (R^2^ 0.91, p < 2.16x10^-16^), suggesting that the updated model should still yield accurate Component 3 score prediction using only 6 trait measures. Predicted Component 3 scores were then incorporated into their respective replication data sets for association analyses to be performed.

Table 2 shows the results of the replication analysis. Association testing of the predicted Component 3 score showed a direction specific trend toward association between rs1396315 and Component 3 in the unrelated Norfolk Island outgroup (P = 0.048). Furthermore, the US replication cohort showed a significant association in the same direction as both the Norfolk Island discovery and outgroup cohorts (p = 1.13x10^-5^, Table 2). Given the tight familial clustering and known founder effects of the discovery cohort it is not surprising that the effect size for both replication cohorts drops substantially (>50%). Clearly, this effect size reduction has impacted on the statistical significance of the associations. Nevertheless, these replication findings provide compelling evidence for a robust association between rs1396315 and Component 3 and suggest a sample size of at least 400 unrelateds are required to detect the effect as statistically significant (P<0.05).

**Table 2:** Genotypic Association Analysis between rs1396315 and Component 3 in an unrelated Outgroup from Norfolk Island and an independent replication cohort.

cohort	n	Ref Allele	MAF	Beta.est	std.error	t stat	P-value	P (one-tailed)
NI (discovery)	330	T	0.246	0.572	0.094	37.223	1.05E-09	-
NI (outgroup)	375	T	0.231	0.182	0.109	1.671	9.56E-02	4.78E-02
CHDWB	738	T	0.230	0.150	0.035	4.264	2.27E-05	1.13E-05

### Gene expression and pathways analysis

**Fig 5:**
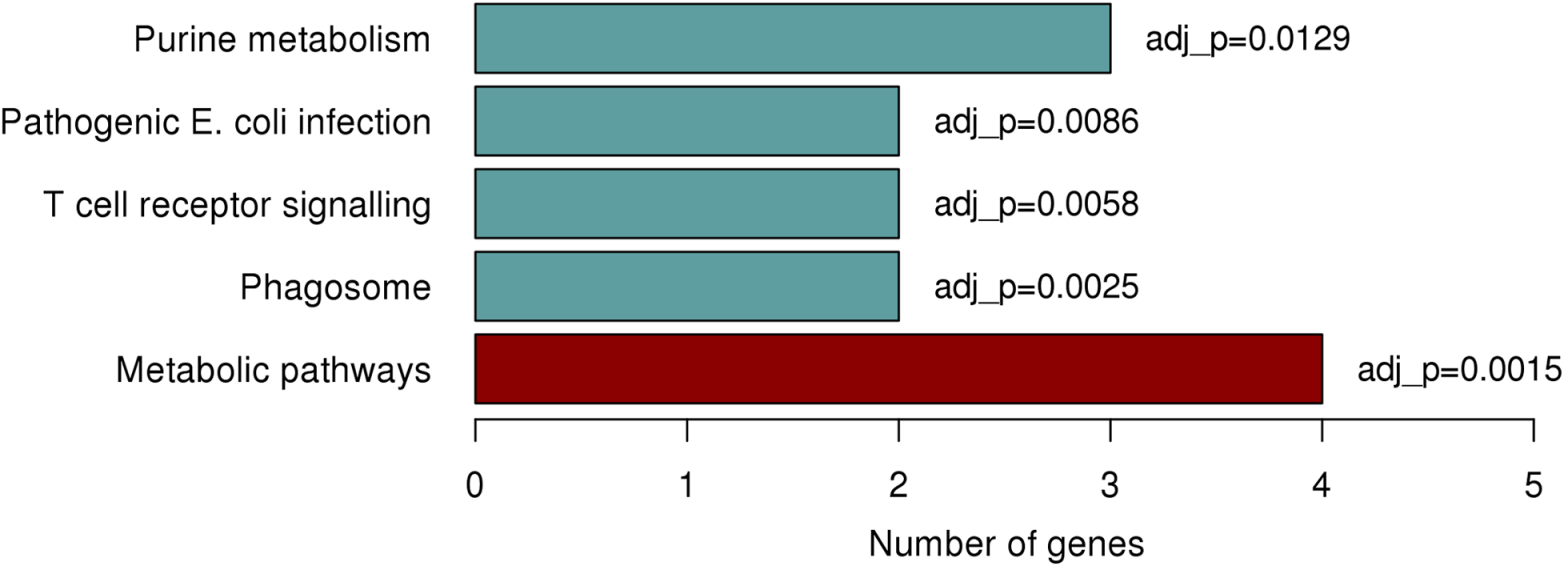
Significantly enriched pathways identified from KEGG enrichment analysis.

Due to the lack of functional genes in close proximity to the identified region on chromosome 1p22.2 we explored the potential of *trans* (distal) associations with the rs1396315 and available gene expression data. We interrogated 1712 genetically heritable expression transcripts previously identified in the Norfolk Island population and measured in the same individuals [22]. Analysis revealed 55 significantly associated (P<0.05) transcripts, with the majority of transcripts showing positive association with this SNP (S3 Fig). When annotated, 35/55 transcripts were assigned to well documented genes of known function, with the remaining 20 residing in regions of the genome that are less well annotated. Gene set enrichment analysis of these 35 genes identified 5 functional pathways exhibiting significant enrichment (Fig 5). The most significantly enriched pathway was found to be that of purine metabolism (P = 0.0015), with 3 genes showing enrichment; *PDE6D*, *GART*, and *NME2*. This finding is interesting as previous work in Norfolk Island reported on a set of eQTL associated genes implicated with CVD-risk traits which also resided with the purine metabolism pathway [22].

## Discussion

Many heritable CVD risk traits tend to be correlated suggesting the presence of underlying genes acting pleiotropically to influence multiple traits. GWASs aimed at composite CVD traits may reveal such genes, which would remain undetected using conventional single phenotype analyses. Analysis of large pedigrees from isolated populations can offer advantages for mapping disease genes including the ability to estimate heritability for disease prioritisation and the possible presence of larger effect loci. Here we conducted a study to search for pleiotropic effect loci associated with CVD risk in the well characterized genetic isolate of Norfolk Island. We performed PCA and heritability testing and identified a heritable component trait strongly loaded with indices of metabolism and kidney function. We subsequently performed pedigree-based GWAS to identify a risk haplotype on chromosome 1p22.2 that explains 11% of the variance in this composite trait. This represents a very large effect exerted by a single locus and may reflect the fundamental importance of pleiotropic genes on this phenotype in this genetic isolate. Because the composite phenotype was not able to be standardised for comparison in external populations and given the unique nature of the Norfolk Island pedigree we could not perform conventional replication analysis, instead using a proxy prediction based approach. Replication analysis in an independent outgroup from Norfolk Island supported the association of rs1396315 and Component 3 (P = 0.048). We also investigated trait/rs1396315 association in a completely independent US cohort and observed significant association between the predicted Component 3 score and rs1396315 (P = 0.000011). Additionally, the clinical relevance of the Component 3 score was explored by comparison with the well-known Framingham CVD risk score [23] which showed a positive correlation indicating that the T allele of rs1396315 is associated with increased risk of CVD in the Norfolk population.

A number of GWAS studies have reported association of loci at chromosome 1p22.2 with CVD risk traits. Zabaneth *et al*., reported association of SNPs in this region to metabolic syndrome (including obesity) in 2554 Indian men [24]. Beck *et al*., reported association to liver enzyme indices in a population of 133,653 Europeans and Asians [25]. Hasstedt *et al*., reported association of chromosome 1p22.2 locus to coronary artery disease in 984 whites [26]. Additionally, several GWAS have identified SNPs within 1p22.2 associated with both obesity related traits [27] and liver enzyme levels (gamma-glutamyl transferase) [28]. Interestingly in another GWAS the same intergenic region of 1p22.2 was associated with atherosclerosis and coronary artery calcification [29].

Our examination of the genomic landscape around the chromosome 1p22.2 region showed this to be a relatively gene-poor region with no obvious candidate genes for CVD risk; the nearest genes are *ZNF326* (zinc finger protein) and *BARHL2* (BarH-like homeobox 2) (Fig 4). We are unaware of any literature indicating an association between CVD and related traits with these genes, however a recent review implicates the role of zinc finger proteins in adipogenesis [30], suggesting that this could be a potential target gene to follow up in further studies. Despite the lack of gene annotation it is quite plausible that the presence of non-protein coding elements, eg. epigenetic marks, *trans*-acting eQTLs, or long-non-coding RNAs within this region may be influencing genes and/or other regulatory factors at a distance.

Following this line of thought, we investigated available annotation, including that from several pertinent UCSC tracks: evofold RNA loop prediction; ENCODE transcription factor binding sites from ChIP-SEQ, and tissue specific RRBS methylation information from liver and kidney (both relevant tissue types for the identified phenotype). This feature based annotation revealed important structures and control regions within, or in close proximity to, the association peak. This includes tissue specific epigenetic markers from kidney and liver, as well as several transcription factor binding sites (S2 Fig). While none of the transcription factor binding sites related to the *trans*-eQTLs explored in our analyses, it is interesting to note that there are several, such as FOXA1, that are directly associated with metabolism in tissues such as the liver. FOXA1 is of particular interest as its family members have been shown to play an important role in the differentiation of the pancreas and liver and regulate metabolism in mice [31]. Additionally, several transcription factor sites annotated directly under the association peak are known pioneer factors, meaning they can bind condensed chromatin and influence transcription and potentially even methylation. We also noted that there were 4 annotated QTL loci that have been mapped from the rat genome to the human genome (S2 Fig). While these QTL are coarsely mapped to the human genome from rat data, it is of particular interest to note that they are all metabolically related, and 2 are directly related to the function of the kidney. All these observations provide additional support for the association of an important pleiotropic site, associated with metabolism and kidney function, at 1p22.2

To investigate potential *trans*-acting mechanisms we conducted an association analysis between the top ranking Component 3 SNPs and heritable gene expression transcripts measured in the same individuals [22]. Pathways analysis of genes encoding transcripts showing significant association with SNPs revealed that the purine metabolism pathway was the most significantly enriched. This is consistent with our previous study which identified enrichment of *NME1* and *PAPPS1* in the purine pathway in the Norfolk Island cohort [22]. In the current study transcripts for *GART*, *NME2* and *PDE6D* were found to be associated with rs1396315. Whereas *NME1* encodes for the A isoform, *NME2* encodes for the B isoform of nucleoside diphosphate kinase (NDPK) which maintains the balance of nucleosides and high energy nucleotides (ATP/GTP) via the reversible transfer of phosphate. Interestingly, GART plays a central role in the *de novo* synthesis of nucleotides where it catalyses three of the ten steps involved in the conversion of phosphoribosyl pyrophosphate into inosine monophosphate [32]. Genetic variation in both *GART* and *NME2* would have the potential to have fundamentally important effects on nucleotide metabolism and homeostasis.

Modifications in the purine metabolic pathway are linked with a number of diseases including kidney-related diseases such as hyperuricaemia and gout [33]. Combined with our previous data there is evidence of a shift in the regulation of purine synthesis and metabolism in the Norfolk Island cohort that may be linked with the development of renal dysfunction and associated CVDs. Variation in *PDE6D* was also identified in relation to the purine pathway. *PDE6D* encodes phospohdiesterase (PDE) 6δ subunit of the enzyme PDE6 that specifically hydrolyzes cyclic GMP [34]. PDE6 is localised in rod or cone membranes of the mammalian retina focussing research on its involvement in visual dysfunction [35]. These findings may provide insight into the high incidence of ocular diseases, including glaucoma, in the Norfolk Island population [36]. Interestingly there is also data indicating that *GART* also plays a role in ocular development [37].

Overall, our findings provide convincing evidence for a significant pleiotropic effect locus at chromosome 1p22.2 associated with a composite trait representing metabolic and/or renal dysfunction. This study illustrates the value of the phenomics approach in large pedigrees and genetic isolates for identifying underlying genetic regulators of inter-correlated disease traits that would go undetected using conventional single phenotype approaches. Further studies are now warranted to better understand the importance of the associations in the broader population and to more comprehensively interrogate the functional relevance of this locus in terms of renal pathology and CVD risk more generally.

## Materials and Methods

### Sample/cohort collection, pedigree information and ethics

The Norfolk Island Health Study (NIHS) is well established [9,12,14,22,38]. The Norfolk Island pedigree structure was previously outlined in Bellis *et al*., 2008 [39], and has recently been updated Macgregor *et al*., 2010 [12]. The updated pedigree structure includes ~5700 Norfolk Island individuals, spanning 11 generations and ~200 years. In this study we focused on a reduced ‘core’ pedigree, meaning that individuals; a) were genetically related to the original founders, and b) had phenotype (S1 Table) and genotype information available. The total number of core pedigree members examined was 330 (excluding individuals under the age of 18) and spans 4 generations at its greatest depth. The Norfolk Island 'outgroup' population used as a validation cohort consisted of 375 individuals. These individuals are all unrelated to the core-pedigree members as inferred by lack of presence in the pedigree, or a genetic kinship of 0. A summary of the available phenotype data for these 375 individuals is available (S3 Table). All individuals gave written informed consent. Ethical approval was granted prior to the commencement of the study by the Griffith University Human Research Ethics Committee (ethical approval no: 1300000485). Ethics approval and management of the NIHS has since been transferred to Queensland University of Technology.

### The US CHDWB replication cohort

The replication cohort in Atlanta is the Center for Health Discovery and Well Being (CHDWB) cohort of the Emory-Georgia Tech Predictive Health Institute [21]. Whole genome genotypes were imputed from either Illumina OmniQuad or Illumina Core+Exome genotyping arrays using Impute2 against the 1000 Genomes. Over 200 trait measures are available for the cohort, at baseline and between 3 and 5 subsequent visits over a 4 year period (n = 738). Participants are all employees of Emory University between the ages of 25 and 75 contacted at random and representing a broad cross-section of the geography of the city of Atlanta and socioeconomic diversity. Summarised clinical data for the seven major traits is available (S4 Table). Written informed consent for participation in genetic research was provided under the auspices of the Institutional Review Boards of Georgia Tech and Emory University.

### Multiple phenotype analysis

Baseline statistics for the core-pedigree (discovery cohort) were calculated for 37 CVD-related measures including all the biochemical measures as well as body size and composition traits (S1 Table). Phenotypic baseline statistics were calculated in SPSS 18.0. All 37 traits were then subjected to Principle Component Analysis (PCA). Briefly, the PCA method is a multivariate reduction method that is commonly used to identify latent components in highly dimensional datasets. When applied to a set of observed variables, PCA will extract a smaller number of artificial variables (principle components) which should account for the majority of the total variance originally observed. Thus one of the major benefits of PCA is that once an underlying set of components has been identified the data can be compressed with very little loss of information. These component scores can then be retained and utilised in further analyses. The experimental method utilised in this study is termed unsupervised in the sense that the PCA was run across all the available CVD trait data, as opposed to selecting a subset of core CVD related traits (such as BMI, weight, lipids, blood pressure, and insulin). All previous published studies have utilised a more ‘supervised’ (selective) approach in this sense, and we hypothesise that this may impact on the outcome of underlying CVD component structures. PCA was conducted on 37 biochemical and anthropometric measures related to CVD. Components with eigenvalues greater than 1 were retained; the number of significant variables loading on a component was reduced through the implementation of orthogonal rotation (varimax). All PCA trait loadings for each component are available (S2 Table).

### Heritability analysis

The genetic analysis program SOLAR [40] was utilised to calculate heritability estimates for all 37 traits as well as the 13 defined principle components. The extended Norfolk pedigree was used by SOLAR to estimate h^2^ (heritability). Both the individual and component traits were screened for covariant effects of age|sex interactions. All traits which SOLAR calculated as having a high kurtosis were log transformed before calculating heritability estimates.

### Genome-wide genotyping

EDTA anticoagulated venous blood samples were collected from all participants. Genomic DNA was extracted from blood buffy coats using standard phenol-chloroform procedures (Qiagen). Genome-wide genotyping was carried out using the Illumina Human610-Quad v1.0 beadchip. Raw data from Illumina idat files was SNP genotyped in R using the CRLMM package [41]. Genotype data then underwent QC routines using PLINK [42]. Briefly, SNP analysis was restricted to autosomal SNPs with minor allele frequency >0.01, call rate >0.95 and Hardy-Weinberg equilibrium testing p-value >10^−5^. After quality control, 590,603 SNPs were used for association analyses. Genotype data was then exported from PLINK and imported into the CRAN package GenABEL [43] for analysis.

### Genome-wide association analysis, LD testing and genotype association

A pedigree based GWAS analysis of the 9 heritable components was batched using custom R scripts and the package GenABEL, using the polygenic model with age and sex interactions, as well as genetic structure (the top 2 principal components of the complete SNP set as calculated by KING [44]). Specifically, the polygenic model function implemented in GenABEL is capable of estimating the narrow sense heritability of a trait (h^2^). The polygenic model takes into account the fact that potentially thousands of genetic variants contribute to a trait phenotype. The function implemented in GenABEL maximises the likelihood of the data under the polygenic model with covariates and reports twice negative maximum likelihood estimates and the inverse of the variance-covariance matrix at the point of maximum likelihood. GenABEL also estimates residuals of the trait and the inverse of the variance-covariance matrix for further use in association analysis with the mmscore function. The polygenic model within GenABEL implements variance components defined to account for linked major gene effects, background polygenic effects, and environmental effects. Age and sex were included as covariates, as well as genetic structure which was assessed by principal components analysis using the KING [44] program. The top two components were chosen as covariates because we found that these explained the majority of the variance in the outcomes being tested and inclusion of additional, less informative, components only served to reduce the parsimony of the models. All heritable transcripts were then treated as phenotypes and batched GWAS were run. The mmscore function as implemented in GenABEL was used. This function represents a mixed model approximation analysis for association between a trait and genetic polymorphism, and is specifically designed for association testing in samples of related individuals. This allows for per SNP association testing using a polygenic (mixed) model approach [43]. The study-wide significance was set based on *M*_*eff*_ adjustment (P = 1.84x10^−7^). Haplotype/LD testing was conducted in Haploview 4.2 [45]. Exploration of the region surrounding the peak on chr 1p22.2 and integration of LD information and UCSC track data was performed using LocusTrack [20] within R.

### Replication analysis

Ideally replication would be conducted on the exact same set of 37 clinical traits, and involve generating component scores using the original coefficients. However, it is extremely difficult to obtain a separate population with the exact same phenotype information, making this option not possible. We ultimately have performed a linear model approach, using the 7 most highly loaded Component 3 traits to predict the score in our replication cohorts, the unrelated Norfolk Island outgroup (n = 375), and the US CHDWB replication cohort (n = 738). Details of the modelling are given in results, but overall the model was constructed from the discovery cohort (NI core-pedigree, n = 330) using the lm (linear models) base R function:

$$fit_{1}=lm\left(COMP3\;\sim \;WHR+BF+SBP+DBP+CREAT+UREA+U\_ACID\right)$$

Predictions were then made using the predict function in base R as follows:

$$COMP3.pred=predict.lm\left(fit_{1},replication.data\right)$$

Association testing of Component 3 (predicted) and rs1396315 in the replication cohorts was performed using linear modelling, with the predicted component score as the outcome and the genotype as the independent variable. The genotype was coded in an additive fashion: 0 (CC); 1 (CT), 2 (TT).

### eQTL association analysis

The generation and list of eQTLs in the Norfolk Island cohort has previously described in detail [22]. Blood from the same n = 330 Norfolk Island core-pedigree samples was collected and stored at -20°C in PAXgene tubes (Qiagen, Valencia, CA). PAXgene Blood miRNA Kits kits (Qiagen) were used to extract total RNA according to the manufacturers’ instructions and RNA was assessed for quality using the Bioanalyzer 2100 (Agilent Technologies, Santa Clara, CA). A total of 250ng RNA was amplified and labelled using the Illumina TotalPrep-96 RNA Amplification Kit (Life Technologies, Grand Island, NY), according to the manufacturers’ instructions. Expression profiling was performed using the Illumina HumanHT-12 v4.0 Expression BeadChip Kit (Illumina, San Diego, CA) using 750ng of amplified RNA and following the Whole-Genome Gene Expression Direct Hybridization Assay Guide. Array images were scanned on the Illumina iScan and analyzed initially using the Gene Expression Module of GenomeStudio (V2011.1). Background subtraction was applied and missing bead types were imputed using GenomeStudio. Based on the number of expressed probes (at “detection p-values” < = 0.05), mean raw expression values across probes, and correlations (across probes) between samples, all samples provided high quality data, except for one sample that was of questionable quality and that was removed. Significantly expressed probes were then determined at a false discovery rate (FDR) of 5%, based on p-values generated in a binomial test on the counts of samples in which a probe generated “detection p-values” < = 0.05 (success) and >0.05 (failure). Subsequently, the raw expression levels of probes detecting significant expression were shifted by a constant amount so that the minimum observed value of any probe in any sample was 1.0, followed by log2 transformation and quantile normalization. A series of custom filters were designed to identify cis/trans eQTL’s; presence of multiple adjacent SNPs in a peak (within +/-20Kb), a quick parse SNP/CHR location filter, a chromosome quadrants filter, and a graphical filter (modified Manhattan Plots with kern smoothing to facilitate peak identification) [22]. All eQTLs were defined by the 'tagging' SNP, the SNP with showed the most significant association with the given transcript. A set of n = 2200 SNPs representing all those within eQTL peaks which surpassed the *M*_*eff*_ adjusted threshold (1.84x10^-7^) were extracted and used as a basis for an eQTL-centric association analysis. This analysis involved Component 3 phenotype being run in a GWAS-based approach in GenABEL [43] using the extracted SNP set. A short list of traits was obtained by using a relaxed significance threshold 1.0x10^-2^.

## Supporting Information

S1 Table Descriptive statistics for 37 traits measured for the Norfolk Island pedigree. (PDF)

S2 Table PCA trait loadings for all 13 extracted components. (PDF)

S3 Table Summarised clinical information for the seven important Component 3 traits in the Norfolk Island outgroup cohort. (PDF)

S4 Table Summarised clinical information for six important Component 3 traits in the US CHDWB replication cohort. (PDF)

S1 Fig Individual trait associations for the 7 clinical traits significantly loaded on Component 3. The red dotted line indicated the study-wide *M*_*eff*_ adjusted threshold (p<1.84x10^-7^).
(PDF)

S2 Fig UCSC based region annotation for chr 1p22.2 association locus, detailing DNase hypersensitivity clusters and transcription factor binding sites. (PDF)

S3 Fig Association of the top 3 Component 3 SNPs with previously identified heritable eQTL transcripts. (PDF)
