# Supplementary material for: A Phenomic Scan of the Norfolk Island Genetic Isolate Identifies a Major Pleiotropic Effect Locus Associated with Metabolic and Renal Disorder Markers
Source: PLoS Genet. 2015 Oct 16;11(10):e1005593. doi: 10.1371/journal.pgen.1005593 (PMC4608754; doi:10.1371/journal.pgen.1005593)
Supplement: S1 Table — (PDF) [file pgen.1005593.s001.pdf]

S1 Table: Descriptive statistics for 37 traits measured for the NI pedigree

Trait	Total (mean)	Std dev	N	Male (mean)	Std dev	N	Female (mean)	Std dev	N
Age	49.38	15.87	330	48.93	15.53	152	49.77	16.20	178
Height	1.70	0.09	329	1.77	0.07	152	1.65	0.06	177
Weight	76.71	16.04	329	86.11	14.10	151	68.73	12.99	178
Wasit Circumference	87.40	13.89	311	95.43	10.77	139	80.91	12.71	172
Hip Circumference	102.09	9.60	311	103.61	7.04	140	100.85	11.13	171
WHR	0.86	0.08	310	0.92	0.06	139	0.80	0.06	171
BMI	26.47	4.62	328	27.58	4.17	151	25.53	4.78	177
Body fat	30.08	8.78	326	24.01	6.51	150	32.26	6.96	176
SBP	130.48	24.32	325	136.32	21.39	151	125.41	25.59	174
DBP	77.06	13.47	325	80.25	13.80	151	74.30	12.58	174
Glucose	5.62	0.96	315	5.77	0.99	147	5.48	0.92	168
Cholesterol	5.64	1.11	330	5.81	1.09	152	5.50	1.11	178
Triglycerides	1.99	1.17	330	2.29	1.23	152	1.72	1.05	178
Chol/HDL ratio	4.32	1.33	327	4.83	1.31	150	3.89	1.19	177
HDL-C	1.38	0.34	327	1.26	0.29	150	1.49	0.35	177
LDL-C	2.85	0.97	329	3.03	0.94	151	2.70	0.97	178
Albumin	41.54	2.73	327	42.11	2.76	151	41.05	2.62	176
Globin	29.64	3.45	327	29.65	3.66	151	29.63	3.28	176
Total protein	71.18	4.66	327	71.75	5.02	151	70.69	4.27	176
LDH	124.46	34.53	327	126.04	31.58	151	123.11	36.91	176
Creatinine	81.29	16.75	326	89.40	16.22	150	74.38	13.88	176
Total Bilirubin	7.59	4.19	327	8.25	4.20	151	7.02	4.10	176
Direct Bilirubin	2.55	1.43	324	2.68	1.47	149	2.45	1.40	175
Alk Phos	63.47	18.34	327	68.07	19.21	151	59.53	16.62	176
GGT	27.97	29.23	327	36.92	34.34	151	20.29	21.26	176
ALT	18.13	11.61	327	21.46	12.15	151	15.27	10.34	176
AST	21.37	9.36	327	23.55	9.54	151	19.50	8.81	176
Uric acid	0.34	0.09	324	0.40	0.08	151	0.30	0.08	173
Sodium	140.35	2.42	326	140.89	2.57	151	139.88	2.18	175
Potassium	4.23	0.70	327	4.22	0.52	151	4.23	0.83	176
Chloride	102.46	17.03	322	101.55	2.66	148	103.23	23.03	174
Bicarbonates	29.07	3.29	327	29.45	2.75	151	28.74	3.66	176
Anions	13.74	3.25	327	14.06	3.61	151	13.47	2.88	176
Urea	5.65	1.50	327	6.07	1.32	151	5.28	1.56	176
Calcium	2.38	0.10	327	2.39	0.01	151	2.37	0.11	176
Phosphate	1.14	0.71	327	1.13	0.75	151	1.16	0.68	176
Iron	16.53	4.98	326	17.29	4.81	150	15.88	5.05	176
