# Supplementary material for: A Phenomic Scan of the Norfolk Island Genetic Isolate Identifies a Major Pleiotropic Effect Locus Associated with Metabolic and Renal Disorder Markers
Source: PLoS Genet. 2015 Oct 16;11(10):e1005593. doi: 10.1371/journal.pgen.1005593 (PMC4608754; doi:10.1371/journal.pgen.1005593)
Supplement: S2 Table — (PDF) [file pgen.1005593.s002.pdf]

Rotated Component Matrix[a]

	Component						
	1	2	3	4	5	6	7
WEIGHT	.753	.015	.274	.225	.363	.108	.077
WC	.746	.182	.333	.243	.324	.067	.067
HIP	.927	.102	.072	.081	.056	.003	-.037
WHR	.303	.188	.443	.304	.446	.094	.133
BMI	.907	.141	.178	.079	.200	-.029	-.043
BF	.642	.101	-.384	-.172	-.301	-.224	-.079
SBP	.379	.324	.559	-.005	-.102	.022	.056
DBP	.411	.335	.408	.038	-.134	.040	.222
CHOL	.104	.928	.099	.119	-.057	-.063	.085
CHOLHDL	.220	.649	.112	.106	.633	-.101	.036
HDL	-.189	-.055	-.040	-.025	-.874	.072	.014
LDL	.059	.939	.034	.064	.024	.039	.044
ALBU	.042	.107	.058	.098	.017	-.019	.791
GLOB	.022	.003	.045	.049	.063	.065	-.016
T_PROT	.040	.066	.072	.095	.055	.043	.450
LDH	.150	.226	.127	.304	-.128	-.021	-.461
CREAT	.040	.059	.742	.158	.185	-.014	.151
T_BILI	.009	-.030	.021	.032	-.022	.884	-.031
D_BILI	-.002	-.201	-.053	.013	-.045	.812	-.183
ALK_PHS	.162	.191	.178	.351	-.012	-.109	-.002
GGT	.071	.157	.135	.642	-.054	.020	.180
ALT	.167	-.046	.057	.758	.223	.107	-.059
AST	.035	.120	.073	.820	.014	.092	.023
U_ACID	.226	.090	.608	.359	.284	.047	.071
SODIUM	-.013	.129	.186	-.022	.114	.110	.145
POTA	-.004	.041	.147	.001	-.017	-.036	.239
CHLOR	-.072	.067	-.044	-.090	.099	-.051	.078
BICARB	-.046	.068	.138	-.076	-.056	.079	.132
ANIONS	.049	.074	.096	.093	.029	-.045	.148
UREA	.166	.056	.757	-.065	-.106	-.132	-.249
CALC	-.003	.096	.064	.088	-.041	-.114	.675
ADJ_AL	-.023	.023	-.026	.050	-.052	-.101	.114
PHOS	.021	-.023	-.112	-.099	.000	-.111	.088
IRON	-.001	.104	-.023	.181	-.069	.560	.207
lipid_risk	.157	.808	.103	.038	.297	-.100	-.043
TRIG_Log	.275	.355	.191	.155	.487	-.222	.027
GLU_Log	.159	.010	.188	.073	.110	-.051	.125

Rotated Component Matrix[a]

	Component				
	8	9	10	11	12
WEIGHT	-.036	-.014	-.055	.046	.099
WC	-.068	-.091	-.009	.115	.154
HIP	.024	.034	.003	.015	.050
WHR	-.126	-.167	-.019	.163	.193
BMI	.014	-.008	-.009	.068	.042
BF	.176	.065	-.058	.025	-.157
SBP	.193	-.261	-.065	.219	-.081
DBP	.185	-.262	-.104	.169	-.090
CHOL	.036	.032	.014	.019	.006
CHOLHDL	.081	.052	-.038	.038	-.082
HDL	-.055	-.054	.058	-.031	.115
LDL	-.033	.061	.031	-.072	.016
ALBU	.221	.109	-.069	-.124	.130
GLOB	.917	-.004	-.010	.083	-.041
T_PROT	.852	.060	-.045	-.010	.043
LDH	.223	.045	.102	-.221	.262
CREAT	-.015	.115	.036	-.047	.028
T_BILI	.063	.028	-.020	.072	-.035
D_BILI	-.044	.019	.116	.047	.051
ALK_PHS	.035	.200	.265	.255	.156
GGT	-.016	-.213	-.017	.292	.078
ALT	.042	.076	-.098	-.113	-.003
AST	.087	.054	-.075	-.001	-.090
U_ACID	.059	.051	.016	.034	-.002
SODIUM	-.028	.739	-.195	-.037	.222
POTA	-.126	-.054	.136	-.686	-.160
CHLOR	.070	-.038	.106	-.112	-.387
BICARB	-.075	.233	.707	-.039	.223
ANIONS	-.011	.168	-.829	.046	.231
UREA	.075	.170	.035	.006	-.109
CALC	.205	.513	.206	-.004	-.146
ADJ_AL	.062	.648	.290	.113	-.366
PHOS	.062	-.054	.103	-.086	.647
IRON	.090	-.064	-.005	-.205	-.077
lipid_risk	.026	.050	-.019	.014	-.110
TRIG_Log	.191	.036	-.026	.221	-.065
GLU_Log	-.028	-.023	.060	.606	-.145

Extraction Method: Principal Component Analysis.
Rotation Method: Varimax with Kaiser Normalization.

a. Rotation converged in 15 iterations.
