# Supplementary material for: A Phenomic Scan of the Norfolk Island Genetic Isolate Identifies a Major Pleiotropic Effect Locus Associated with Metabolic and Renal Disorder Markers
Source: PLoS Genet. 2015 Oct 16;11(10):e1005593. doi: 10.1371/journal.pgen.1005593 (PMC4608754; doi:10.1371/journal.pgen.1005593)
Supplement: S3 Table — (PDF) [file pgen.1005593.s003.pdf]

NI_outgroup

S3 Table: Summarised information for the 7 important component 3 traits in the Norfolk Island outgroup cohort.

trait	total (mean)	std dev	n	male (mean)	std dev	n	female (mean)	std dev	n
WHR	0.88	0.08	375	0.93	0.07	156	0.84	0.07	219
BF	32.74	8.63	375	27.16	7.20	156	36.72	7.28	219
SBP	135.00	25.37	375	140.00	23.30	156	131.00	26.14	219
DBP	83.00	16.10	375	86.00	14.33	156	82.00	17.02	219
CREAT	81.34	17.76	375	90.53	16.97	156	74.80	15.26	219
UREA	5.70	1.70	375	5.93	1.48	156	5.53	1.82	219
U_ACID	0.40	0.19	375	0.50	0.15	156	0.33	0.18	219
