# Supplementary material for: A Phenomic Scan of the Norfolk Island Genetic Isolate Identifies a Major Pleiotropic Effect Locus Associated with Metabolic and Renal Disorder Markers
Source: PLoS Genet. 2015 Oct 16;11(10):e1005593. doi: 10.1371/journal.pgen.1005593 (PMC4608754; doi:10.1371/journal.pgen.1005593)
Supplement: S4 Table — (PDF) [file pgen.1005593.s004.pdf]

US_replication

S4 Table: Summarised clinical information for the 7 important component 3 traits in the US replication cohort.

trait	total (mean)	std dev	n	male (mean)	std dev	n	female (mean)	std dev	n
WHR	0.82	0.10	738	0.91	0.07	271	0.77	0.07	467
BF	31.72	9.00	738	24.59	5.88	271	35.85	7.83	467
SBP	118.43	15.56	738	120.01	13.92	271	117.51	16.39	467
DBP	75.11	10.54	738	78.35	9.51	271	73.23	10.66	467
CREAT	0.84	0.14	738	0.96	0.11	271	0.77	0.11	467
UREA	5.26	1.42	738	5.80	1.26	271	4.95	1.42	467
