# Supplementary figures and images for: A Phenomic Scan of the Norfolk Island Genetic Isolate Identifies a Major Pleiotropic Effect Locus Associated with Metabolic and Renal Disorder Markers
Source: PLoS Genet. 2015 Oct 16;11(10):e1005593. doi: 10.1371/journal.pgen.1005593 (PMC4608754; doi:10.1371/journal.pgen.1005593)

**S1 Figure:** GWAS Manhattan plots for the 7 traits loaded on component 3.

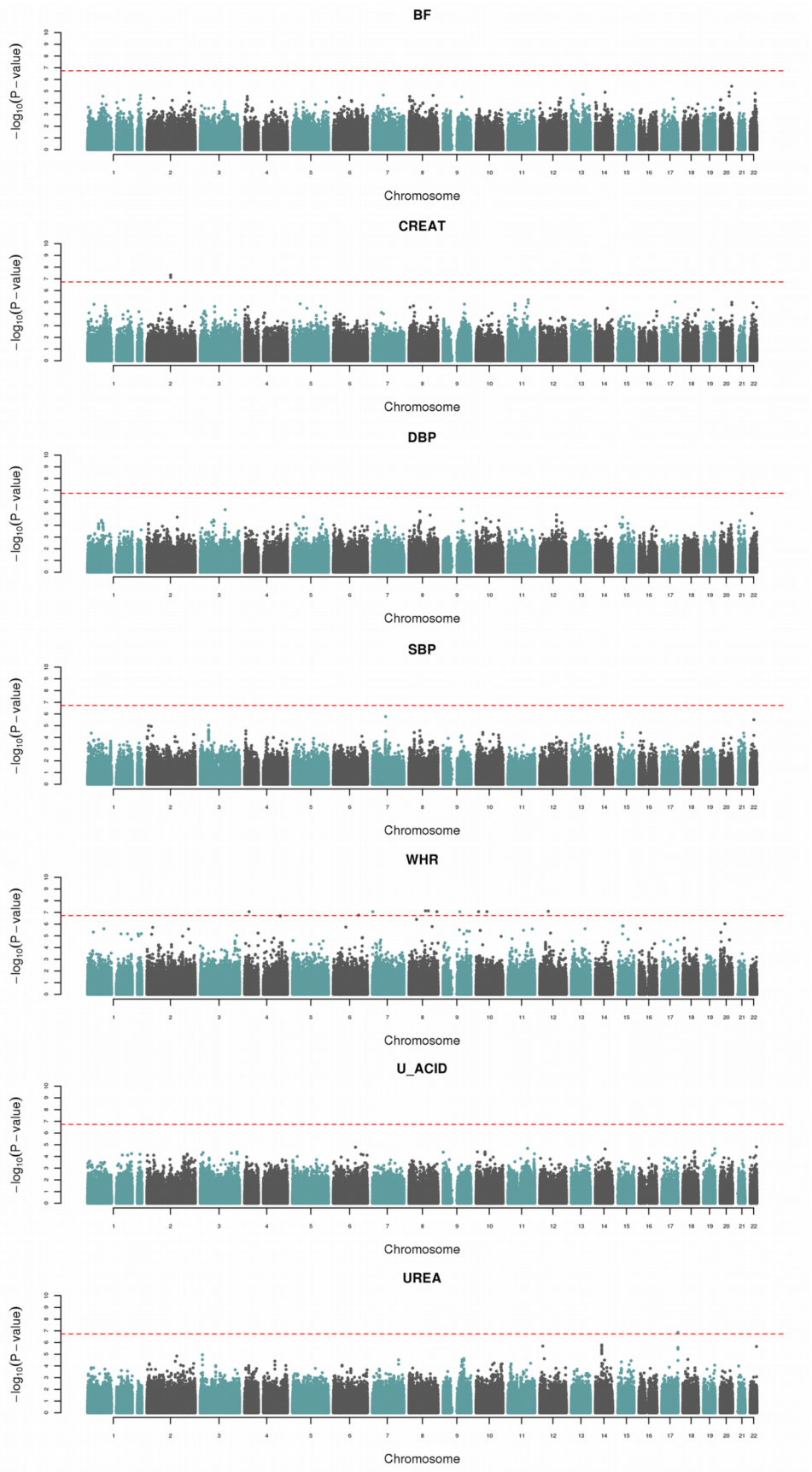

Supplement: S1 Fig — The red dotted line indicated the study-wide M eff adjusted threshold (p<1.84x10^-7^). (PDF) [file pgen.1005593.s005.pdf]

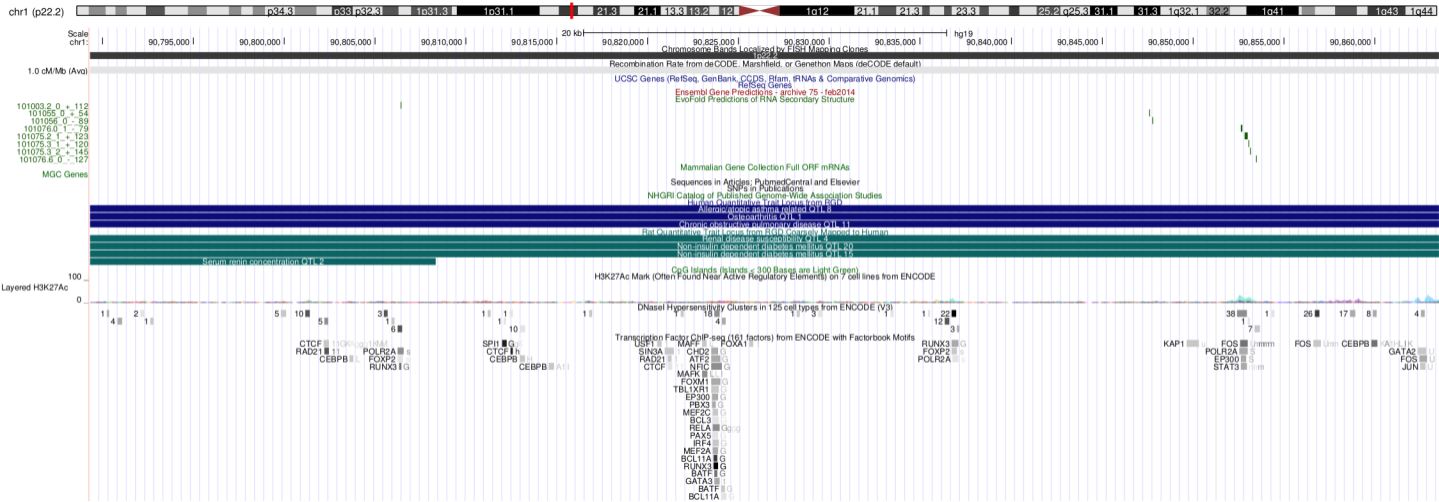

Supplement: S2 Fig — (PDF) [file pgen.1005593.s006.pdf]
