# Supplementary material for: A Phenomic Scan of the Norfolk Island Genetic Isolate Identifies a Major Pleiotropic Effect Locus Associated with Metabolic and Renal Disorder Markers
Source: PLoS Genet. 2015 Oct 16;11(10):e1005593. doi: 10.1371/journal.pgen.1005593 (PMC4608754; doi:10.1371/journal.pgen.1005593)

S3 Figure: Association of top 3 Component 3 SNPs with previously identified heritable eQTL transcripts.

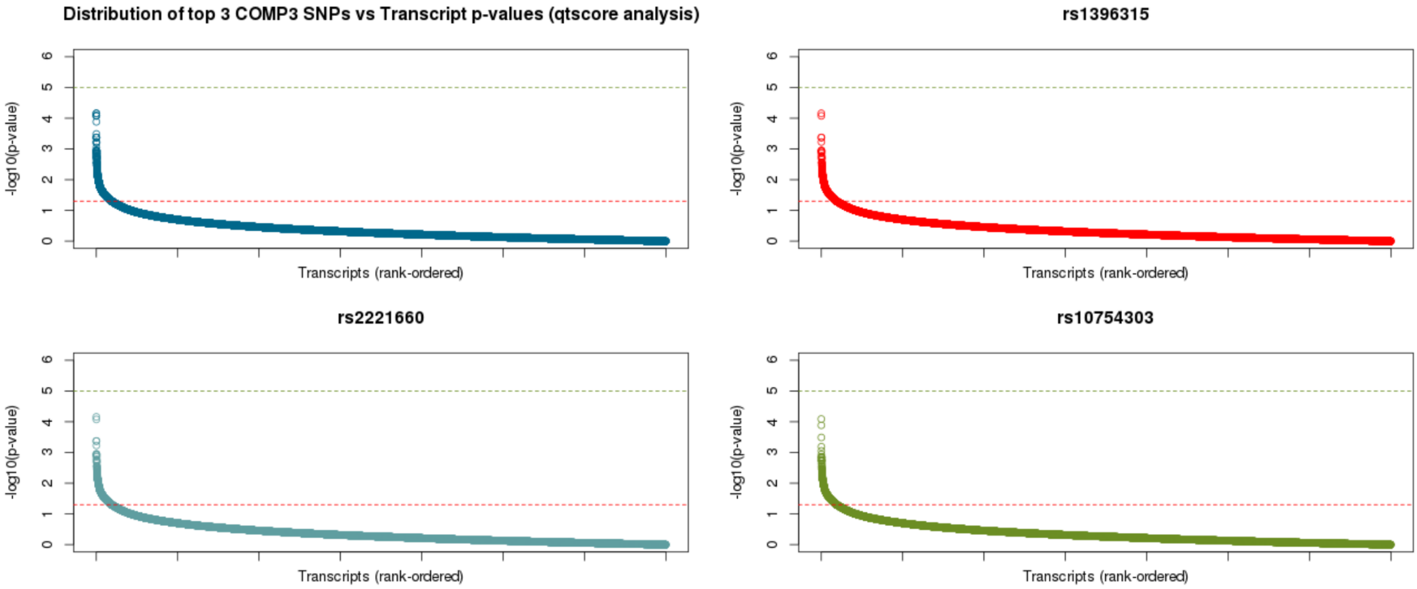

Supplement: S3 Fig — (PDF) [file pgen.1005593.s007.pdf]
